# CENPW knockdown inhibits progression of bladder cancer through inducing cell cycle arrest and apoptosis

**DOI:** 10.7150/jca.90449

**Published:** 2024-01-01

**Authors:** Peng Zhang, Qian Yang, Xulong Chen, Xiaolong Chen, Qing Wang, Kun Chen, Yu An, Kehua Jiang, Fa Sun

**Affiliations:** 1 Guizhou Medical University, Guiyang, China.; 2 Department of Urology, Guizhou Provincial People's Hospital, Guiyang, China.; 3 Department of Gastroenterology, Guizhou Provincial People's Hospital, Guiyang, China.; 4 Department of Medical Genetics, Guizhou Provincial People's Hospital, Guiyang, China.; 5 NHC Key Laboratory of Pulmonary Immune-Related Diseases, Guizhou Provincial People's Hospital, Guiyang, China.

**Keywords:** Bladder cancer, Disease-specific survival, Progression, Cell cycle, Apoptosis

## Abstract

**Purpose:** The objective of this study was to examine the expression and role of Centromere protein W (CENPW) in bladder cancer (BLCA), as well as its potential mechanistic impact on the progression of BLCA.

**Methods:** In this study, we conducted a comparative analysis of the mRNA expression level of CENPW in BLCA tissues and adjacent normal tissues using data from the Cancer Genome Atlas (TCGA) and Gene Expression Omnibus (GEO) databases. Additionally, we investigated the association between CENPW expression and patient prognosis. Furthermore, we performed *in vitro* and* in vivo* experiments to assess the impact of CENPW knockdown on various tumor biological phenotypes in BLCA. Finally, we conducted an analysis to elucidate the underlying mechanisms responsible for the observed phenotypic alterations in BLCA.

**Results:** The expression of CENPW was found to be upregulated in BLCA, and its higher expression was associated with a poorer disease-specific survival (DSS). CENPW was found to have close associations with the cell cycle, mitosis, and DNA replication. *In vitro* and *in vivo* experiments demonstrated that the inhibition of CENPW led to a suppression of BLCA progression. Specifically, the knockdown of CENPW resulted in cell cycle arrest phase and induced apoptosis in BLCA by potentially inactivating the signal transducer and activator of transcription3 (STAT3) signaling pathway.

**Conclusion:** CENPW has the potential to function as a molecular marker indicating an unfavorable prognosis in BLCA. Additionally, CENPW exhibits promise as a novel therapeutic target for BLCA.

## Introduction

Based on the 2020 global cancer statistics data, bladder cancer (BLCA) holds the tenth and thirteenth positions among 36 cancer types in terms of incidence and mortality, respectively 1. In China, it is estimated that there were 91,893 new cases and 42,973 deaths attributed to bladder cancer in 2019, making it the second most prevalent malignancy within the urological system 2. Approximately 90 percent of BLCA cases are urothelial carcinomas, which can be further categorized as either superficial or muscle-invasive types 3. Patients with BLCA may experience trans-urethral resection or radical cystectomy with urinary diversion due to frequent recurrence and rapid progression, leading to significant impacts on their quality of life and socioeconomic status 4, 5.

The advancement of high-throughput sequencing technology and bioinformatics has facilitated the identification of certain proteins that serve as potential biomarkers for cancer prognosis and therapeutic targets 6. Consequently, the identification of oncogenes associated with BLCA holds considerable significance in the diagnosis and treatment of this disease.

Centromere protein W (CENPW), previously referred to as cancer upregulated gene 2 (CUG2), exhibits significant upregulation in various cancer types such as ovarian, liver, colon, and lung cancers, and is implicated in tumorigenesis 7, 8. Notably, CENPW has been recognized as a novel centromeric component essential for the accurate functioning of kinetochores during cellular division 9-11. Previous investigations have demonstrated the potential of centromere proteins as targeted therapies for mitosis-related antitumor interventions 12-14. Besides, CENPW involve in centromere microtubule attachment and meiotic progression of mouse oocytes 15. Therefore, we hypothesized that CENPW might be involved in the mitosis of tumor cells. Nevertheless, the relationship between CENPW and BLCA has not been explored thus far.

This study aims to examine the correlation between CENPW expression and clinicopathological characteristics of BLCA. Additionally, it was discovered that inhibiting CENPW resulted in the suppression of BLCA progression both in vitro and in vivo. Collectively, these findings provide evidence that CENPW could serve as a promising prognostic biomarker and therapeutic target for BLCA.

## Materials and Methods

### CENPW expression in pan-cancer

The unified and standardized pan-cancer datasets can be downloaded from UCSC: TCGA TARGET GTEx (PANCAN, N = 19131, G = 60499). Within this dataset, we specifically extracted information regarding the expression of the CENPW gene in 13 human solid tumors. To analyze the data, we utilized R software (version 3.6.4) to calculate the expression differences between paired tumor and adjacent normal tissues. Furthermore, we employed an unpaired Wilcoxon Rank Sum and Signed Rank Test to assess the statistical significance of these differences.

### Characteristics of CENPW in bladder cancer

The Cancer Genome Atlas (TCGA) dataset was initially employed to identify differentially expressed genes (DEGs) in BLCA compared to normal tissues. To validate the mRNA expression in BLCA, microarray gene expression profiles of bladder urothelial carcinoma (UCB) tumor tissues and adjacent non-tumor tissues were obtained from the Gene Expression Omnibus (GEO) database (GSE13507) 16. The data processing and analysis were performed using Perl (5.30.1) and R (version 3.6.2) software. Two-group comparisons were conducted using either the unpaired Student's t-test or paired t-test. In this study, we conducted an analysis of the relationship between CENPW expression and DSS in two cohorts, namely TCGA-BLCA and GSE13507. To determine the optimal threshold for CENPW expression in BLCA, we employed the "maxstat" R package. Additionally, we utilized the "survival" R package to assess the prognostic significance difference between the two groups using the logrank test method.

### Gene set enrichment analysis and Gene correlation analysis

Furthermore, we investigated the associations of CENPW with biological processes and signaling pathway gene sets using Gene set Enrichment Analysis (GSEA v4.2.1) 17. Significant enrichment was operationally defined as the identification of KEGG pathways with false discovery rate (FDR) q-values less than 0.05 and an absolute normalized enrichment score (NES) greater than 1. Our research specifically focused on investigating the signaling pathways implicated in the development and progression of bladder cancer. Additionally, we conducted gene correlation analysis between the CENPW gene and the enriched genes using the GEPIA2 database 18.

### Tissue specimens

A total of 14 fresh BLCA tissues and corresponding normal tissues were obtained from the Guizhou Provincial People's Hospital, with written informed consent provided by all patients. The inclusion and exclusion criteria for this study encompassed the diagnosis of bladder urothelial carcinoma in all pathological results, the absence of distant metastasis prior to surgery, and the absence of any concurrent malignancies. Detailed patient characteristics pertaining to the tissue samples can be found in **Table S1**. The Institutional Research Ethics Committee of Guizhou Provincial People's Hospital approved this study (NO.2023-072).

### Cells and transfection

The human bladder urothelial carcinoma cell lines (5637, T24, RT4, J82, UM-UC-3) and an immortalized urothelial cell line (SV-HUC-1) were obtained from Procell Life Technology Co. Ltd (Wuhan, China). 10% fetal bovine serum (FBS) was added to RPMI 1640 medium to culture 5637 cells. The T24 and RT4 cells were cultured in Myc medium supplemented with 10% FBS. The J82 and UM-UC-3 cells were cultured in MEM medium supplemented with 10% FBS (Sijiqing, Hangzhou, China). All cells were incubated in a humidified air atmosphere at 37°C. The cells were transfected when they reached a confluence of 60-80%. Transfections with siRNA (GenePharma Co., Ltd.; Shanghai, China) were performed using Lipofectamine RNAiMAX reagent (Introgen, USA) following the manufacturer's instructions. After transfection, the cells were utilized for subsequent experiments within a time frame of 24-72 hours. Stable cell lines were generated using lentivirus and the lentiviral vector pHBLV-U6-MCS-CMV-ZsGreen-PGK-PURO obtained from Hanheng Biotechnology (Hanheng Biotechnology Co., Ltd., Shanghai, China). Puromycin was added to the cell pools at a concentration of 1mg/mL for a duration of 1 week. The knockdown efficiency was assessed through Western blot analysis and RT-qPCR.

### RNA preparation and qRT-PCR

Total RNA was isolated following the manufacturer's instructions of the Total RNA Kit (YEASEN, Shanghai, China). The concentration and purity of the RNA were determined using a Nanodrop 2000 spectrophotometer (Thermo Fisher Scientific). cDNA was synthesized using the Hifair® Ⅲ RT SuperMix for qPCR (YEASEN, Shanghai, China), and the Hieff UNICON® SYBR-Green qPCR master mix (YEASEN, Shanghai, China) was employed for the qPCR. Real-time PCR analysis was conducted using the Bio-Rad CFX Connect Real-Time PCR Detection System. To standardize the measurement of gene expression, the β-actin gene was employed. The primer sequences utilized can be found in **Table S2**. The relative mRNA expression was determined using the 2(^-ΔΔCT^) method and adjusted based on the levels of β-actin mRNA.

### Western blotting

A western blot assay was performed using protein extracted from cultured cells and tissues. Protein samples were separated using 10% and 12% SDS-PAGE and subsequently transferred onto PVDF membranes with a pore size of 0.22μm. The membrane was blocked using 5% skim milk and a primary antibody was then incubated overnight at 4 °C on the membranes. The primary antibodies were used as follows: anti-CENPW (1:1,000, 1030382-3; Abcam, United Kingdom), anti-βactin (1:10,000; 60004-1-Ig; Proteintech, Wuhan, China), anti-Cleavedcaspase 3 (1:1,000, #9661S; Zenbio, Chengdu, China), anti-CyclinB1 (1:1,000, 60186-1-Ig; Proteintech, Wuhan, China), anti-CDK1 (1:1,000, 11554-1-AP; Proteintech, Wuhan, China), anti-Bcl-2 (1:1,000, #2872T; CST, United States), anti-P21 (1:1,000, 50599-2-Ig; Proteintech, Wuhan, China), anti-Bax (1:1,000, 50599-2-Ig; Proteintech, Wuhan, China), and anti-STAT3 (1:1,000, #9139; CST, United States), anti-p-STAT3 (Tyr705) (1:2000, #9145; CST, United States). A chemiluminescence system was employed to detect protein bands subsequent to the incubation of primary antibody probes with secondary antibodies. The scanned blots were subjected to analysis using ImageJ version 1.51 in order to ascertain the density of the bands. Protein expression was normalized by comparing it to the expression of β-actin.

### Cell proliferation assay

Following a 24-hour transfection with siRNA-CENPW, 5637 and UM-UC3 cells were seeded onto a 96-well plate at a density of 3,000 cells per well. Subsequently, 10μl of cell counting kit-8 (CCK-8) solution (Apexbio, K1018, USA) was added to each well at 24, 48, 72, and 96 hours. The optical density (OD) value for each well was measured at 450 nm after a 2-hour incubation at 37°C using a microplate reader (BioTek, Winooski, VT, USA).

### Edu staining

5637 and UM-UC3 cells were transfected with siRNA-CENPW as previously described. After plating in a 96-well plate for 72 hours, cell proliferation staining was conducted using an Edu kit (C10310-1, RIBOBIO, EDU-567, Guangzhou, China) following the manufacturer's instructions. Cells were imaged using Olympus IX71 inverted fluorescence microscopes, and the quantification of Edu-positive cells was performed using ImageJ (ImageJ 1.52a, United States).

### Clone formation assay

Stable knockdown CENPW 5637 and UM-UC3 cells were seeded onto a 6-well plate (400 cells/well) and incubated for 14 days. Subsequently, the cells were treated with a 4% formaldehyde solution for a duration of 15 minutes. Subsequently, the cells were subjected to staining using a 0.1% crystal violet solution for a duration of 30 minutes and counted. Finally, the colonies were quantified using ImageJ software.

### Immunofluorescence

Frozen tissue sections (8umm thick) or cell sheets were fixed using a 4% paraformaldehyde solution, followed by permeabilization using 0.2% Triton X-100 for 10 minutes. Subsequently, blocking was performed using a 5% bovine serum albumin (BSA) solution from Sigma for a duration of 2 hours at room temperature. The primary antibodies were incubated overnight at 4 °C, followed by treatment with a fluorescein-conjugated anti-rabbit secondary antibody and Tubulin Tracker Green (Beyotime Biotechnology, Shanghai, China) were incubated for 1 hour at room temperature. After a 10-minute incubation period with DAPI, the fluorescence signal was visualized using either an Olympus IX70 fluorescence microscope (Olympus, Japan) or a confocal imaging system (Zeiss, Germany).

### Cell cycle and apoptosis analysis

Following 48 hours of transfection, the cells were harvested through centrifugation at 1000 rpm for 4 minutes. The cells were then fixed overnight at 4°C in 70% ethanol. After 24 hours of fixation, the cells were washed three times with PBS. Subsequently, the cells were stained with a solution containing 500 μl of propidium iodide (Elabscience, Wuhan, China) and RNase A at a ratio of 9:1. The cells were subjected to flow cytometric analysis using a flow cytometer (BD Biosciences, San Jose, CA, United States) following a 45-minute incubation at room temperature. After 72 hours of transfection, the cells were harvested by centrifugation (1000 rpm, 4 minutes) and washed thrice with PBS. Subsequently, the cells were resuspended in 500μl of binding buffer and double stained with Annexin V and PI, in accordance with the instructions provided by the FITC Annexin V/PI Apoptosis kit (Elabscience, Wuhan, China). Flow cytometry was then performed on the samples using the flow cytometer (BD Biosciences) after a 15-minute incubation in the dark at room temperature.

### TUNEL analysis

Prior to analysis, the tumor xenografts were fixed and converted into frozen sections, which were subsequently washed twice with PBS for 5 minutes each time to remove OCT. The apoptosis of the tumor xenografts was assessed using the TUNEL kit (Elabscience, Wuhan, China) according to the standard protocol. Finally, the fluorescence signal of sh-NC and sh-CENPW was observed using a fluorescent microscope.

### Animal models

Eight 4-week-old BALB/c nude mice, obtained from Vital River Laboratory Animal Technology Co, were used in xenograft experiments. All experiments were conducted at the animal center of Guizhou Medical University and were approved by the Animal Care and Use Committee (2305079). The mice were randomly assigned to two groups of four, and 1 × 10^7^ 5637 cells in 200 μl saline were injected subcutaneously into the right armpit of each BALB/c nude mouse. Tumor measurements were taken every third day, starting on the fifth day after injection. The calculation of tumor volume was determined using the formula V = (length x width^2) / 2. Once the tumor xenografts met the experimental requirements, the nude mice were euthanized and the tumor xenografts were excised and imaged.

### Statistical analysis

Statistical analyses were conducted using IBM SPSS Statistics Version 25.0, R Statistical Software Version 3.6.1, and GraphPad Prism 7 (GraphPad Software, CA, United States). A two-tailed paired or unpaired t-student t-test was employed to compare two groups when variables were normally distributed, while a Mann-Whitney U-test was used for other variables. A two-way analysis of variance (ANOVA) with Sidak's test was employed for conducting multiple comparisons. Pearson's correlation analysis was utilized to examine gene correlations. Statistical significance was determined when the* P*-values were below 0.05 (*P < 0.05; **P < 0.01; ***P < 0.001; ****P < 0.0001).

## Results

### CENPW expression in Pan-Cancer

**Figure 1:**
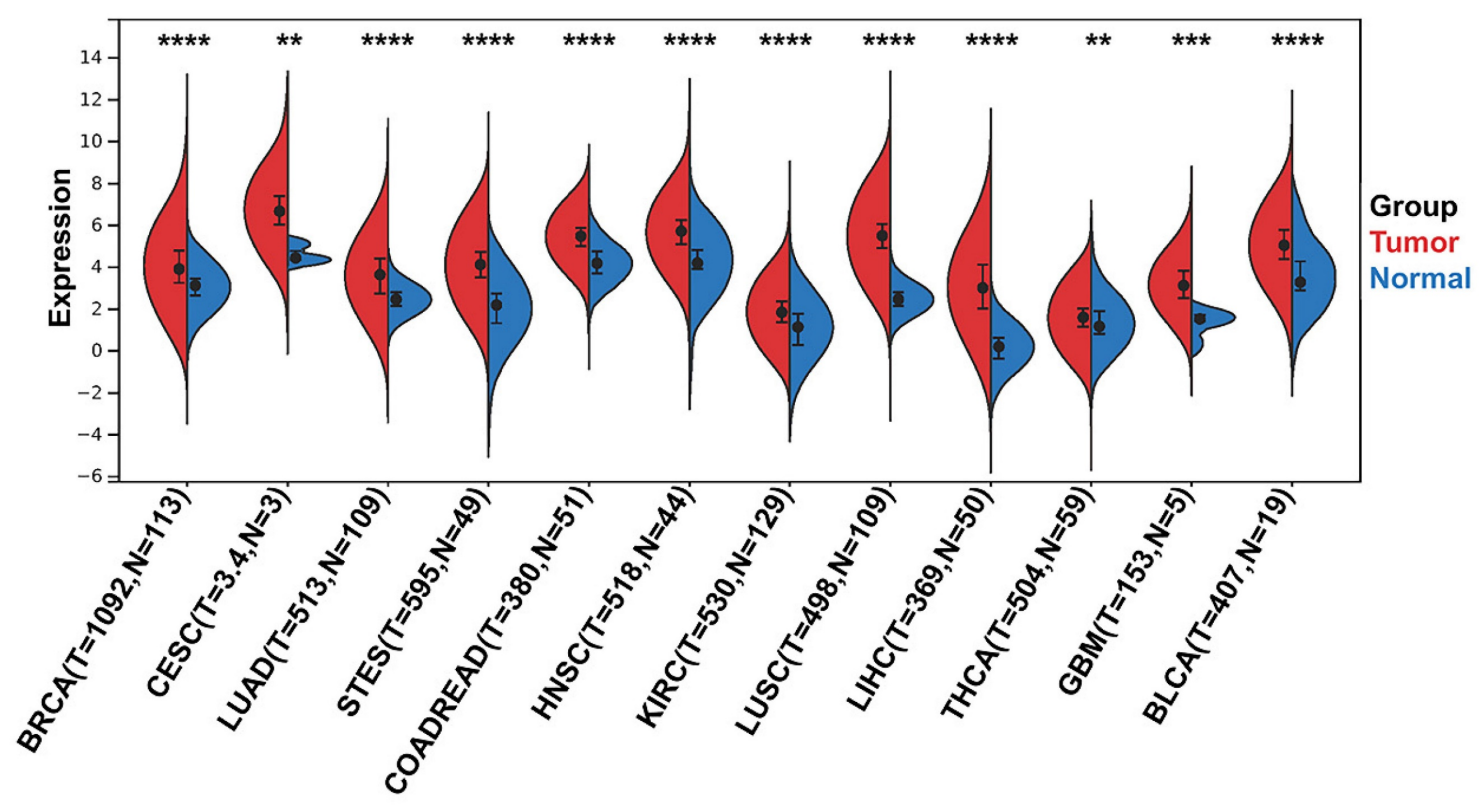
CENPW expression in pan-cancer. Differential CENPW expression analysis in human tumors and normal tissues in UCSC database.

To identify differentially expressed CENPW genes between human tumors and normal tissues, a differential gene expression analysis was conducted using the TCGA and GTEx databases. The findings revealed that CENPW exhibited significantly higher expression levels in breast cancer, cervical cancer, lung cancer, stomach cancer, esophageal cancer, colon cancer, rectum cancer, head and neck squamous cell carcinoma, kidney renal clear cell carcinoma, liver hepatocellular carcinoma, thyroid carcinoma, glioblastoma multiforme, and bladder urothelial carcinoma compared to their respective normal tissues (**Figure 1**).

### Characteristics of CENPW in bladder cancer

**Figure 2:**
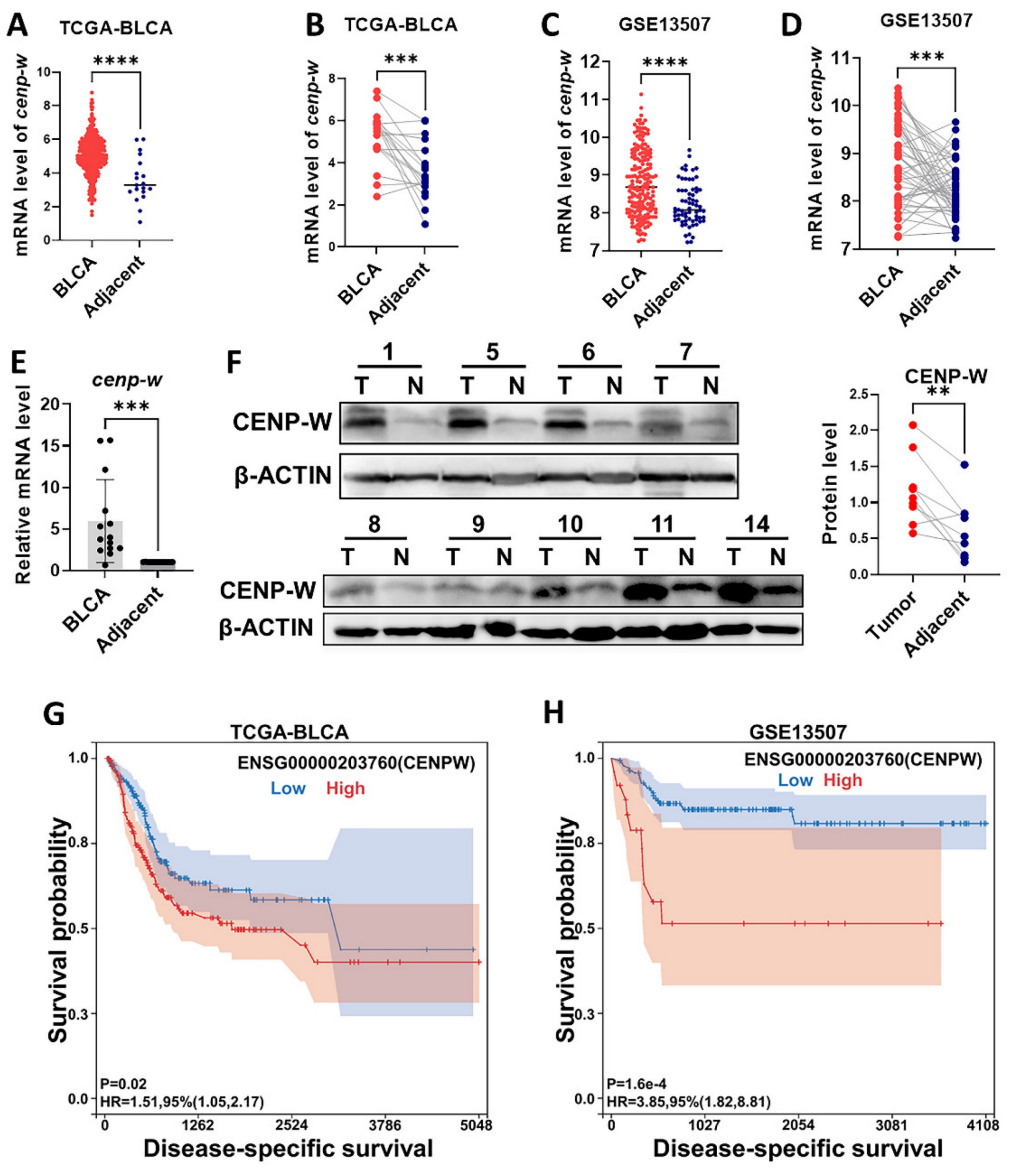
Features of CENPW expression in BLCA. (A, B). CENPW expression in TCGA-BLCA cases, unpaired and paired samples. (C, D). CENPW expression in GSE13507 cases, unpaired and paired samples. (E, F). CENPW mRNA and protein levels in BLCA tissues. (G, H). High CENPW expression is associated with significantly poor DSS.

In order to investigate the biological functions of CENPW in BLCA, we conducted an analysis of both the TCGA-BLCA RNA-Seq databases and the GEO-BUC Gene-chip databases to assess CENPW expression. As anticipated, CENPW exhibited upregulation in BLCA tissues when compared to normal controls in TCGA (407 BLCA tissues versus 19 normal adjacent tissues, *P* < 0.001; 19 pairs of tumor tissues and normal adjacent tissues, *P* < 0.001; **Figure 2A, B**). As CENPW was also found to be upregulated in BLCA tissues compared to adjacent tissues in the GSE13507 cohort from GEO, these findings were consistent with the results obtained from the TCGA dataset (188 BLCA tissues versus 167 adjacent tissues, *P* < 0.0001; 51 matched tumor tissues and adjacent tissues,* P* < 0.001; **Figuer2 C, D**). To confirm the expression of CENPW in BLCA, we assessed both mRNA and protein expression levels in 14 pairs of samples include bladder cancer tissues and normal adjacent tissues. In comparison to adjacent normal tissues, tumor tissues exhibited significantly elevated levels of CENPW mRNA in 14 pairs of tissues (*P* < 0.001; **Figure 2E**). Western blot results demonstrated higher levels of CENPW expression in BLCA tissues compared to adjacent normal tissues in 8 pairs of samples (*P* < 0.01; **Figure 2F**). Furthermore, a notable disparity DSS was observed between patients exhibiting high CENPW expression and those with lower CENPW expression in the TCGA-BLCA dataset (n=385, Hazard Ratio (HR) = 1.51, *P* (HR) = 0.02; **Figure 2G**). Subsequently, we validated the significant correlation between high CENPW expression and poorer DSS in BLCA patients within the GSE13507 cohort (n=165, HR = 4.13, *P* (HR) = 6.4e-5; **Figure 2H**), which aligns with the findings from the PrognoScan database 19.

### Pathway enrichment analysis of genes co-expressed with CENPW

**Figure 3:**
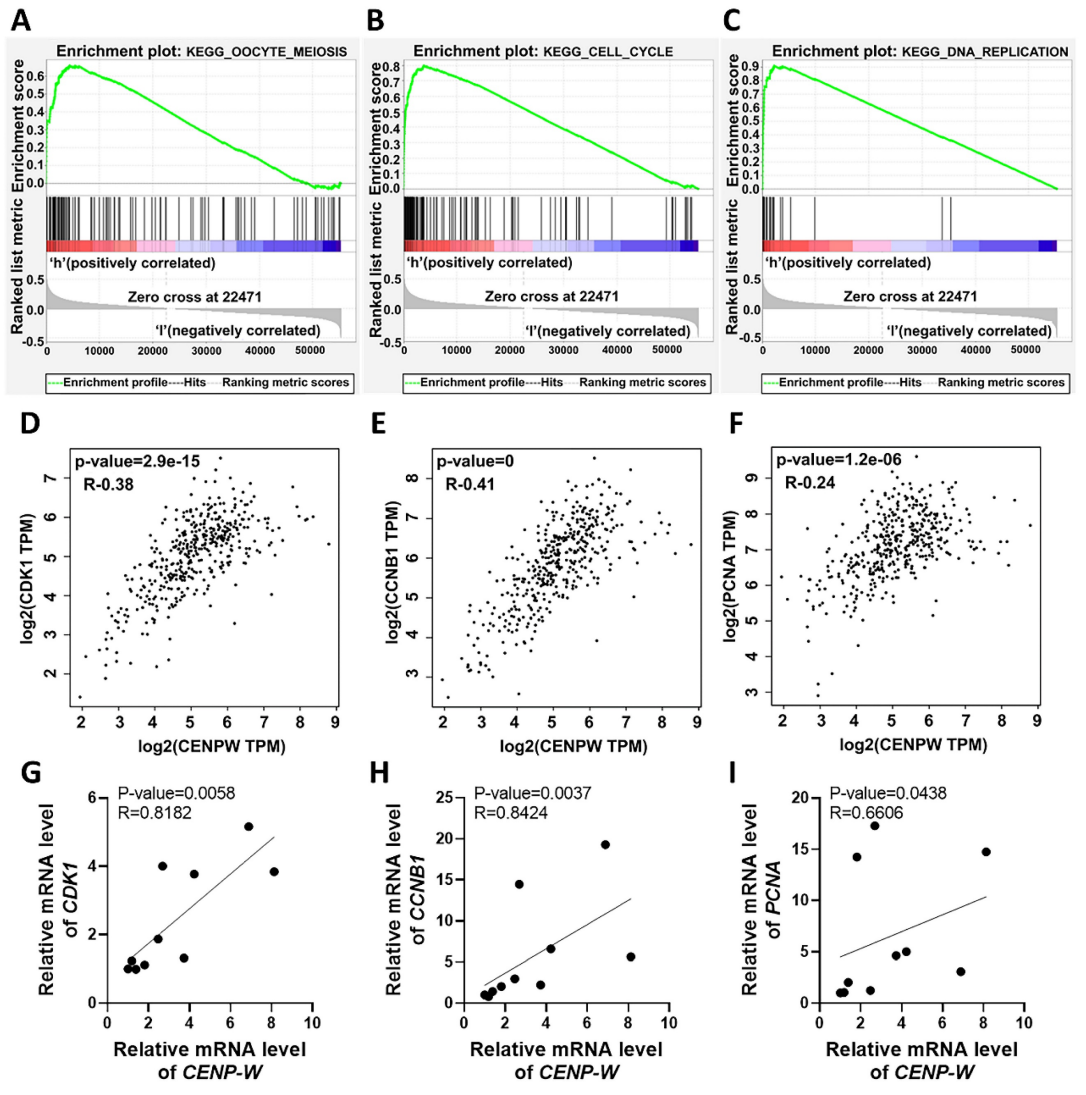
Upregulation of CENPW correlates with activation of cell cycle, mitosis and DNA replication in BLCA. (A-C). Gene set enrichment analysis (GSEA) of CENPW in BLCA. (D-F). Functional gene correlation analysis of CENPW with CDK1, CyclinB1 and PCNA. (G-I). Verified the correlation between CENPW and CDK1, CCNB1 and PCNA expression in bladder cancer samples.

To explore the potential signaling pathways affected by differential CENPW expression in BLCA, we conducted Gene Set Enrichment Analysis (GSEA) on high and low expression CENPW datasets. The analysis revealed that the cell cycle-related pathways (normalized enrichment score [NES] = 2.58, nominal [NOM]-*P* < 0.0001, false discover rate [FDR]-q < 0.0001), oocyte meiosis (NES = 2.38, NOM-*P* < 0.0001, FDR-q < 0.0001), and DNA replication (NES = 2.25, NOM-P =0, FDR-q= 5.022008E-4) were significantly enriched in the high CENPW expression dataset (**Figure 3A-C**). Subsequently, an examination of the associations between CENPW and cell cycle as well as cell proliferation genes was carried out using GEPIA. The findings demonstrated a positive correlation between CENPW gene expression and CDK1 (R=0.38, *P* =2.9e-15), CCNB1 (R=0.41, *P* <0.0001), and PCNA (R=0.24, *P* =1.2e-06) (**Figure 3D-F**). Additionally, the correlation between CENPW expression and CDK1, CCNB1, and PCNA was further investigated in 10 bladder cancer tissue samples. The results consistently indicated a positive correlation between CENPW expression and CDK1 (R=0.8182, *P* =0.0058), CCNB1 (R=0.8424, *P* =0.0037), and PCNA [R=0.6606, *P* =0.0438] (**Figure 3G-I**). The high expression of CENPW might relate to the development of BLCA.

### Knockdown of CENPW inhibits cell proliferation and cell migration

**Figure 4:**
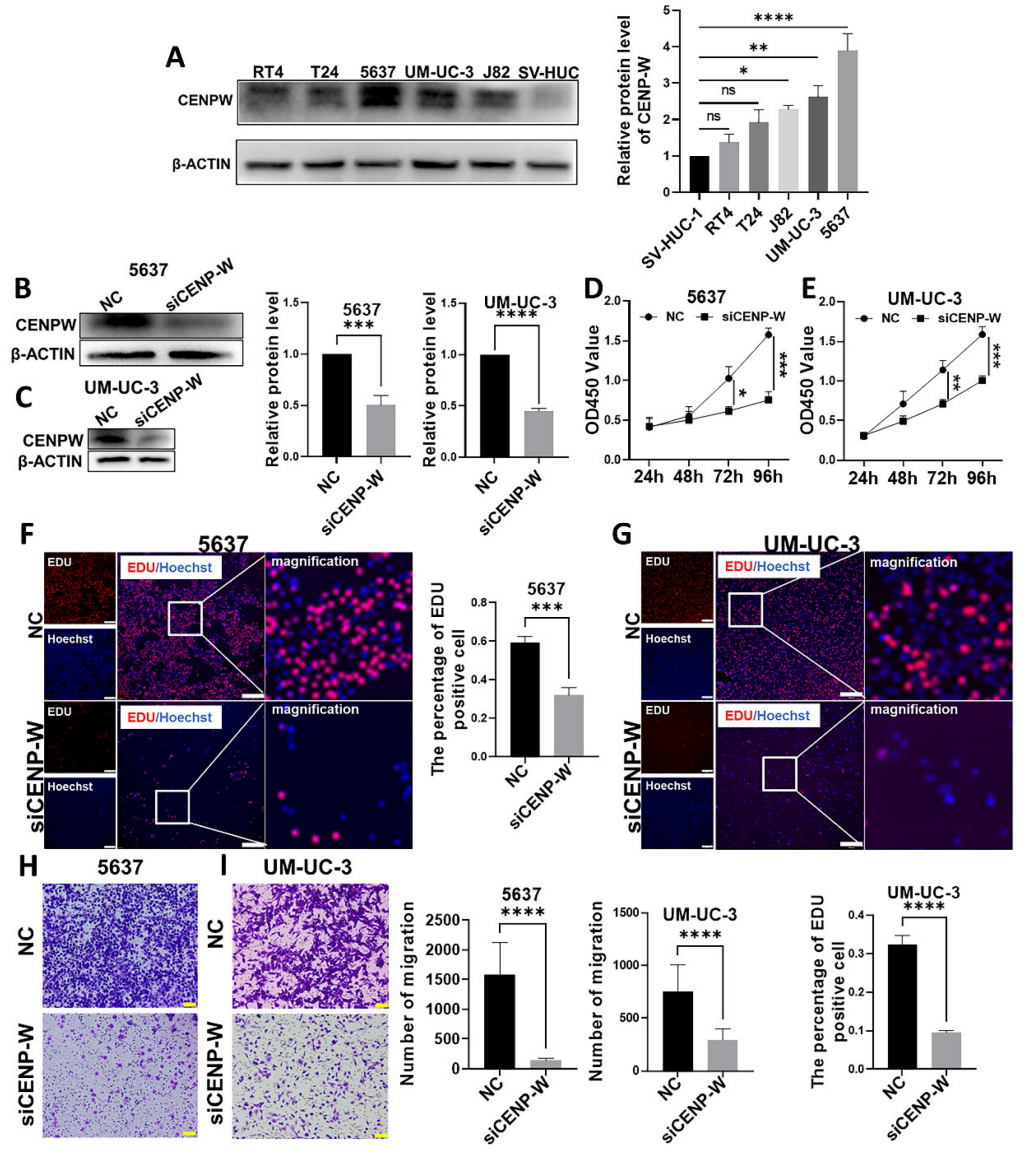
Knockdown of CENPW inhibited bladder cancer cell progression. A. CENPW expression in Urothelial cell lines and bladder urothelial carcinoma cell lines. (B, C). Verification of the efficiency of knockdown of CENPW in 5637 and UM-UC-3 cells. (D-G). Depletion of CENPW inhibited bladder cancer cell proliferation. Bars, 500μm. (H, I). Depletion of CENPW inhabited bladder cancer cell migration. Bars, 100μm.

Initially, the expression of CENPW was examined in normal urothelial cells SV-HUC-1 and bladder urothelial cancer cells (RT4, T24, J82, UM-UC-3, 5637). It was observed that CENPW exhibited significantly higher expression levels in 5637 (*P* < 0.0001), UM-UC-3 (*P* < 0.01), and J82 (*P* < 0.05) compared to SV-HUC-1 (**Figure 4A**). T24 and RT4 did not show a significant difference in CENPW expression compared to SV-HUC-1. Subsequently, 5637 and UM-UC-3 were selected for further loss-of-function experiments. The knockdown efficiency was assessed after sequential transfection of siRNA, revealing a significant decrease in CENPW protein expression in si-CENPW transfected cells (5637, *P* < 0.001; UM-UC-3, *P* < 0.0001) (**Figure 4B, C**). The findings from the CCK8 assay demonstrate a significant reduction in the proliferation capacity of 5637 and UM-UC-3 cells following CENPW knockdown (5637, *P* < 0.001, UM-UC-3, *P* < 0.001) (**Figure 4D, E**). Additionally, the si-CENPW group of 5637 and UM-UC-3 cells exhibited abnormal cell morphology 96 hours after transfection (**Figure S1**). The EDU results further indicate a significant decrease in DNA replication ability in the si-CENPW group compared to the control group (5637, *P* < 0.001, UM-UC-3, *P* < 0.0001) (**Figure 4F, G**). Moreover, the results of cell migration demonstrate a significant decrease in the migration ability of the si-CENPW group compared to the control group. (5637, *P* < 0.001, UM-UC-3, *P* < 0.0001) (**Figure 4H, I**).

### Knockdown of CENPW arrests the cell cycle and induces cell apoptosis

**Figure 5:**
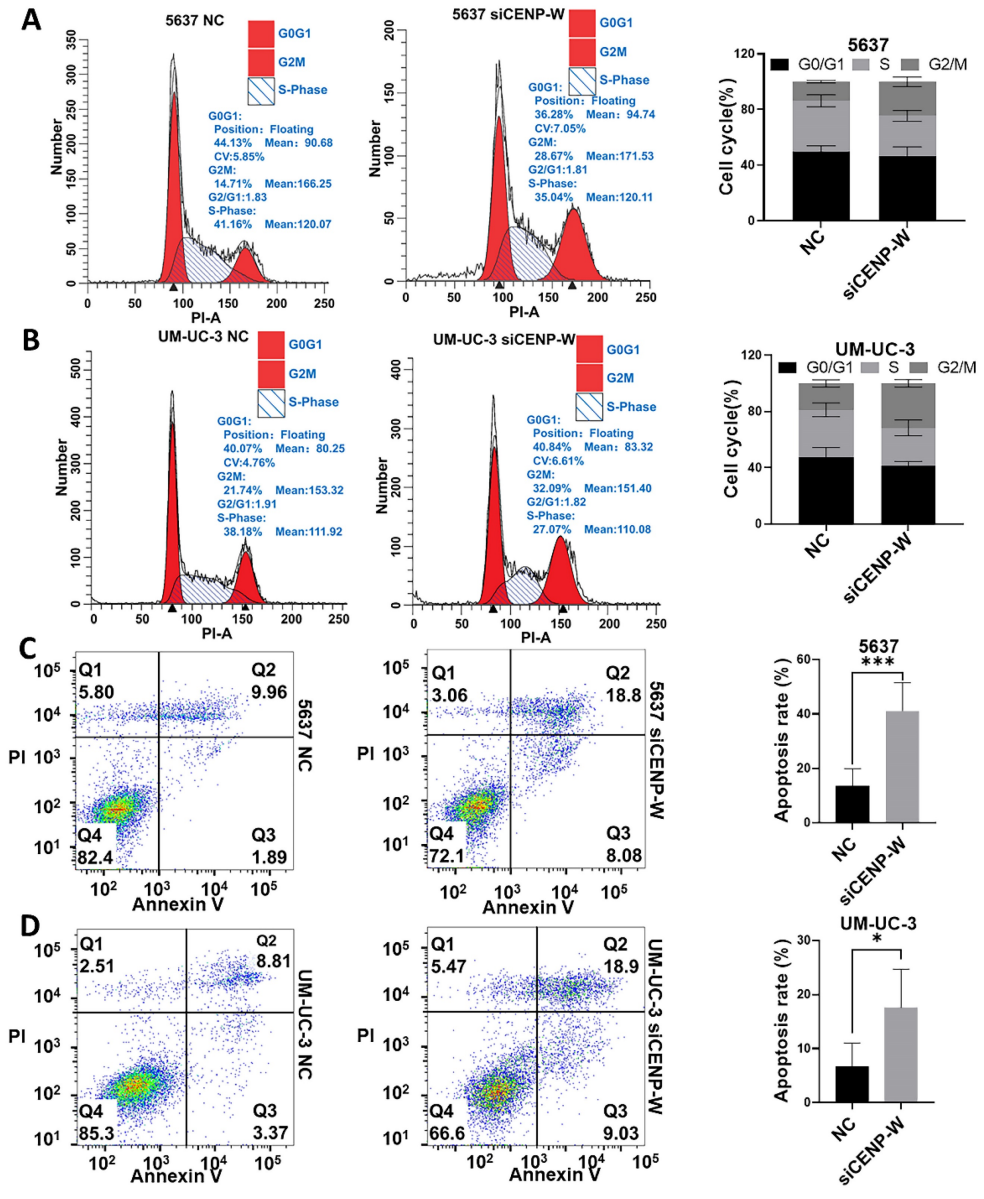
Knockdown of CENPW arrested cell cycle and induced apoptosis. (A, B). Cell cycle phase distribution in si-NC and si-CENPW group of 5637 and UM-UC-3 cells. (C, D). The percentage of apoptotic cells in si-NC and si-CENPW group of 5637 and UM-UC-3 cells.

The results of the cell cycle assay demonstrated that silencing of CENPW led to a significant increase in the proportions of G2/M phase in 5637 (24.53 ± 3.455%, *P* = 0.0009) and UM-UC-3 (31.65 ± 2.554%, *P* = 0.003) cells, compared to the 5637 and UM-UC-3 NC group where the percentages were (13.70 ± 0.9087%) and (18.85 ± 2.503%) respectively (**Figure 5A, B**). Following transfection for 72 hours, the apoptosis rates of 5637 and UM-UC-3 cells were measured and compared between the NC group and the si-CENPW group. The results demonstrated that the apoptosis rates of 5637 and UM-UC-3 cells in the NC group were 13.6 ± 6.3% and6.76% ± 4.29%, respectively. Conversely, in the si-CENPW group, the apoptosis rates were 41.2 ± 10.32% and 17.62% ± 7.07% for 5637 and UM-UC-3 cells, respectively. Notably, transfection with si-CENPW significantly increased the apoptosis rates in both 5637 (*P* < 0.001) and UM-UC-3 (*P* < 0.05) cells (**Figure 5C, D**). Apoptosis tests further revealed that the knockdown of CENPW induced apoptosis in BLCA cells.

### Knockdown of CENPW suppresses tumorigenicity of BCLA cells *in vitro* and *in vivo*

**Figure 6:**
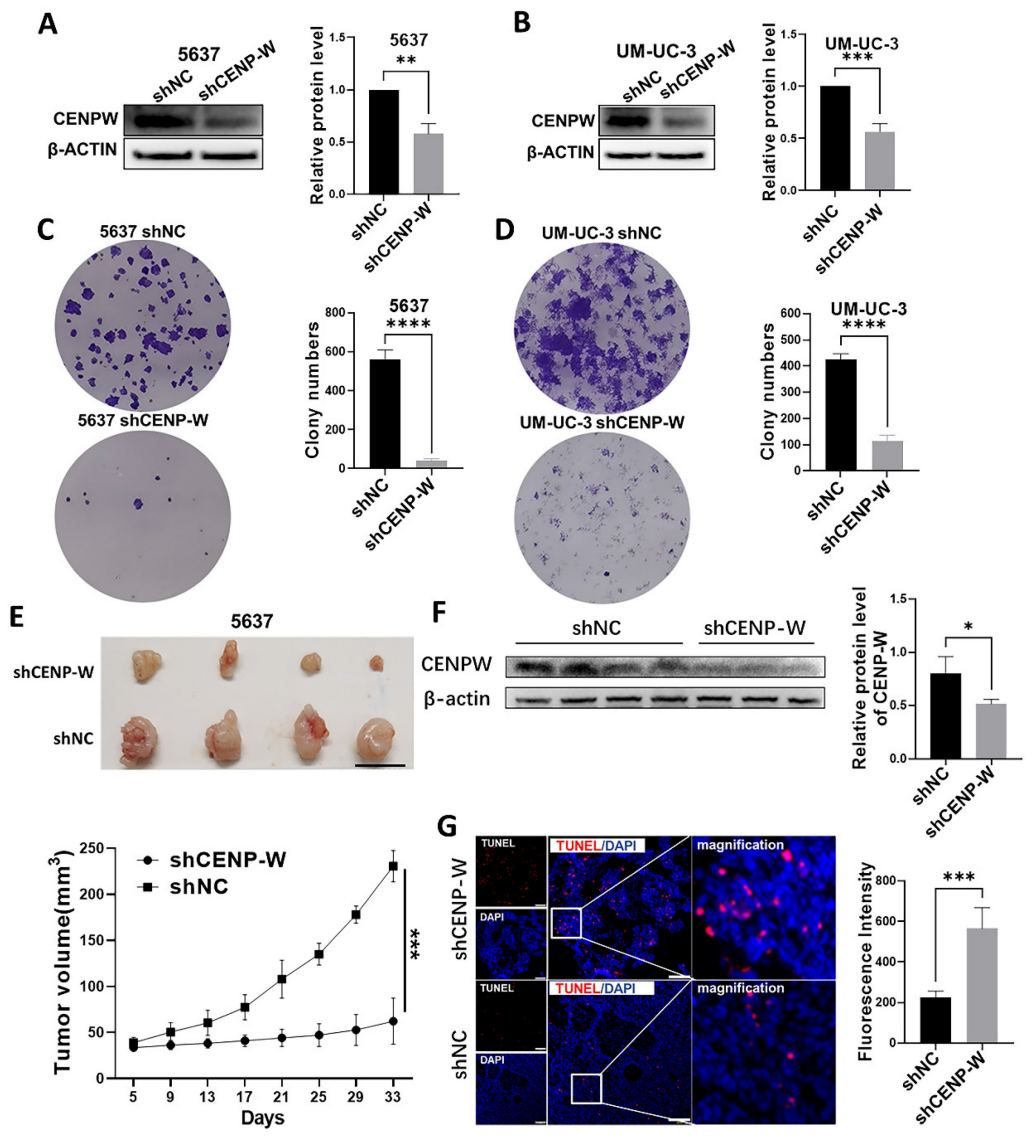
The tumorigenicity was reduced in stable CENPW-knockdown cell lines of 5637 and um-uc-3. (A, B). Verification of the efficiency of knockdown of CENPW in stable cell lines. (C, D). Poor clonogenicity in stable CENPW-knockdown cell lines. E. Weak tumor formation of stable CENPW-knockdown 5637 cell in nude mice. Bars, 1cm. F. The expression of CENPW was decreased in shCENP-W group. G. The higher proportion of apoptosis in the subcutaneous tumor of shCENP-W group. Bars, 100μm.

To assess the long-term effects of CENPW knockdown on BLCA cells, stable knockdown cell lines were established using 5637 and UM-UC-3 cells. The findings of this study indicate that CENPW protein levels was decreased in the sh-CENPW group compared to the sh-NC group (5637 *P* < 0.01, UM-UC-3* P* < 0.001; **Figure 6A, B**). Furthermore, the knockdown of CENPW resulted in a significant reduction in both colony number and size in 5637 (*P* < 0.0001) and UM-UC-3 (*P* < 0.0001) cells, as compared to the sh-NC group (**Figure 6C, D**). In order to investigate the potential antitumor effects of CENPW knockdown in vivo, the tumor volume was measured every 3 days and a tumor growth curve was plotted. The results demonstrated that the tumor growth in the sh-CENPW group was significantly suppressed in comparison to the sh-NC group (*P* < 0.001;** Figure 6E**). The Western blot (WB) analysis provided further confirmation that the protein level of CENPW in the sh-CENPW group was significantly lower than that in the sh-NC group (*P* < 0.05; **Figure 6F**). To investigate the underlying mechanism of tumor inhibition, we conducted a TUNEL assay on frozen sections of the tumors. The red fluorescence intensity of the sh-CENPW group (565.4 ± 102.1) was significantly higher compared to the sh-NC group (224.8 ± 31.39), indicating an increase in apoptosis in the sh-CENPW group (**Figure 6G**; *P* < 0.001).

### Knocking down CENPW inactivates the STAT3 pathway

**Figure 7:**
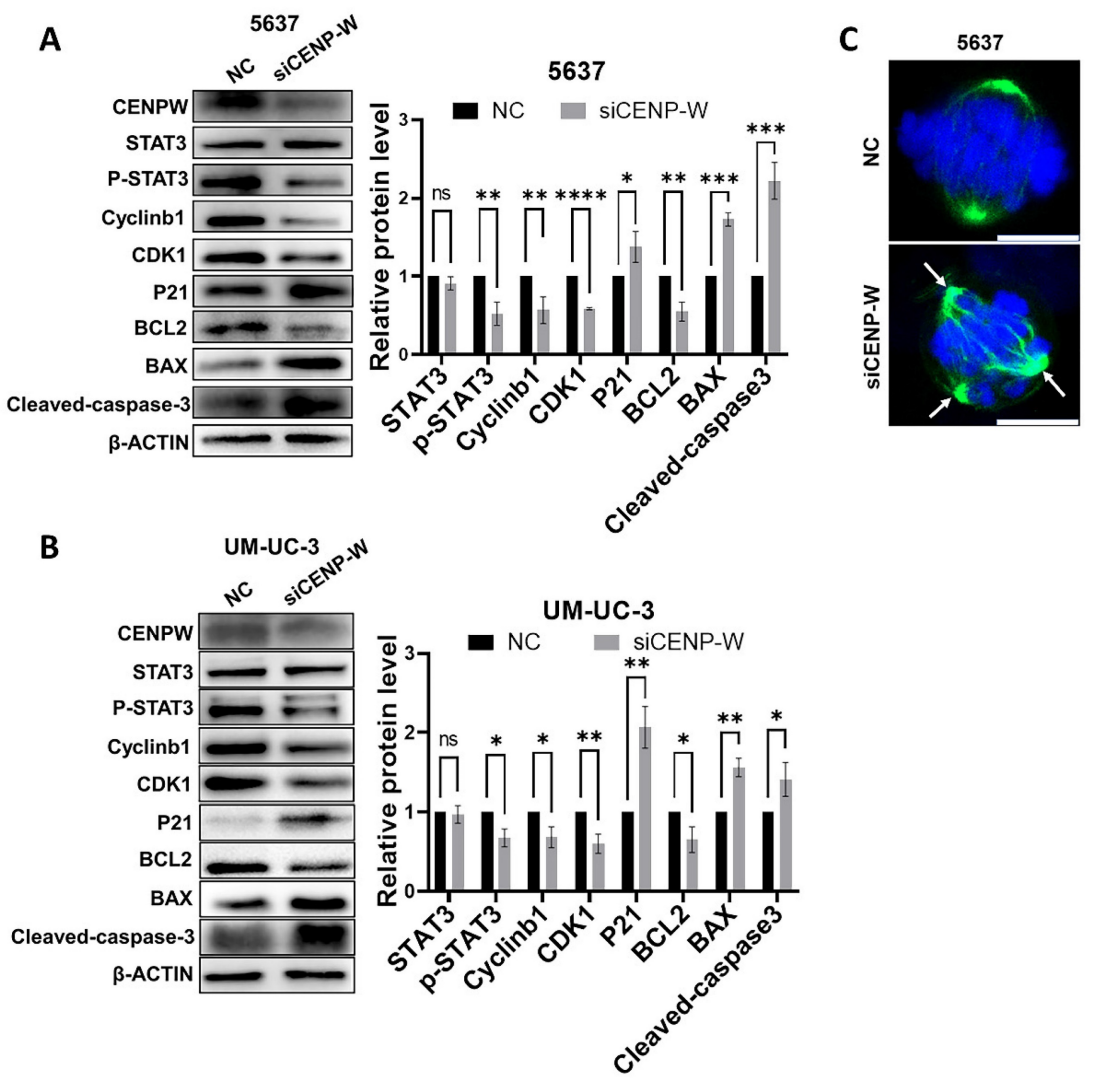
Potential mechanism to inhibit the development of bladder cancer cell. (A, B). Alteration in cell cycle arrest proteins, apoptosis-promoting proteins and apoptosis-inhibiting proteins. C. Abnormal spindle and misaligned chromosomes (white arrow) in si-CENPW group. Bars, 10μm.

To further investigate the mechanism by which CENPW knockdown inhibits bladder cancer, we conducted a comparative analysis of the protein levels of STAT3 and P-STAT3 in both the si-NC group and si-CENPW group of 5637 and UM-UC-3 cells. The results suggest that the depletion of CENPW did not have an impact on the overall protein levels of STAT3 in both the si-NC and si-CENPW groups of 5637 and UM-UC-3 cells. However, the knockdown of CENPW did result in a decrease in the phosphorylated protein level of STAT3 (p-STAT3) in both the si-NC and si-CENPW group of 5637 and UM-UC-3 cells. The expression of cell cycle-promoting proteins CyclinB1 and CDK1, as well as the apoptosis suppressor protein Bcl2, was found to be inhibited compared to the si-NC group. Furthermore, the knockdown of CENPW resulted in an increase in cyclin inhibition protein P21 and the pro-apoptotic protein BAX, as well as Cleaved-caspase-3 in the si-CENPW group (**Figure 7A, B**). Therefore, we suggest that knockdown of CENPW inhibits bladder cancer probably by repressing phosphorylation of STAT3. Additionally, abnormal mitotic cases, such as multi-polarization of spindles and hypercondensed chromosomes, were observed in the si-CENPW group of 5637 cells (**Figure 7C**).

## Discussion

BLCA is categorized into non-muscle-invasive and muscle-invasive types, each exhibiting distinct biological characteristics 20. Approximately 40% of patients with non-muscle-invasive bladder cancer do not exhibit a response to BCG treatment, with some even progressing to muscle-invasive bladder cancer 21, 22. Furthermore, even among those who undergo cystectomy, a subset of patients with muscle-invasive bladder cancer still experience metastasis and recurrence 23. Despite significant advancements in neoadjuvant therapies for bladder cancer, such as immunotherapy, antibody-drug conjugates, and chemotherapy, their efficacy remains limited 24. The search for novel therapeutic targets in the treatment of bladder cancer has been ongoing. Recent research suggests that centromeric protein molecules associated with mitosis could potentially serve as a promising avenue for tumor treatment 12, 25. Specifically, CENPW, a member of the CCNA involved in mitosis, plays a crucial role in ensuring the accurate assembly of sister chromosomes prior to their division. Previous studies have demonstrated its high expression in various cancers, indicating its involvement in promoting cancer progression and resistance 7, 9, 15, 26.

The current study employed the TCGA-BLCA sequencing database and the GSE13507 gene chip database to examine the upregulation of CENPW in BLCA. Our findings demonstrate a significant elevation in CENPW expression levels within BLCA tissues compared to normal bladder tissues, a result that was further validated using frozen tissue samples from bladder cancer patients. Moreover, patients with high CENPW expression exhibited poorer disease-specific survival rates in comparison to those with low expression levels. Furthermore, functional gene analysis revealed a positive correlation between CENPW expression and cell proliferation and cell cycle genes in BLCA. The expression level of CENPW was assessed in 5637, UM-UC-3, J82, T24, RT4 cells, and SV-HUC-1. The results indicated that CENPW expression was significantly higher in BLCA urothelial cells compared to normal urothelial cells. These findings suggest that CENPW may serve as a potential prognostic biomarker for BLCA and contribute to its development.

In this study, transient interference and stable knockdown approaches were employed to treat BLCA cells. The expression of CENPW was notably reduced in both the si-CENPW and sh-CENPW groups when compared to the control group. A series of functional experiments conducted on BLCA cells provided evidence of a close association between CENPW and the progression of bladder cancer. The si-CENPW group exhibited significant reductions in cell proliferation, DNA synthesis, and cell migration when compared to the si-NC group. Additionally, an increasing number of cells in the si-CENPW and sh-CENPW groups displayed morphological changes indicative of apoptosis. To elucidate the underlying cause of the decreased cell proliferation capacity, we analyzed alterations in cell cycle and apoptosis. The results indicated a blockade of the cell cycle at the G2/M phase 27. CDK1 and CyclinB1are known to be factors that promote the cell cycle, and their abnormal expression can disrupt the progression of the G2/M phase of the cell cycle 28. Conversely, P21 is a protein that inhibits the cell cycle by deactivating CDK1, leading to cell cycle arrest 29. Additionally, BCL2 and BAX play roles in regulating apoptosis, with BAX promoting apoptosis and BCL2 inhibiting it. Therefore, we conducted further analysis on the expression levels of proteins related to the cell cycle and apoptosis. Our findings revealed a decrease in the expression of CyclinB1, CDK1, and BCL2 in the si-CENPW group, while the expression of P21, BAX, and Cleaved-caspase3 increased. However, the specific mechanism by which CENPW knockdown influences cell cycle arrest remains unclear. we conducted a detailed analysis of mitosis in both the si-NC and si-CENPW groups. Interestingly, we observed an abnormal formation of the spindle during cell division in the si-CENPW group of 5637 cells. This observation aligns with a previous study which demonstrated that the depletion of CENPW in mouse oocytes led to spindles with abnormal poles during the division phase and activated the spindle assembly checkpoint (SAC) 15. Previous research had shown that tumor cells multi-polarization leaded to cell cycle arrest and apoptosis 30. Based on these findings, we propose that CENPW plays a crucial role in the proper formation of the spindle in BLCA cells. Consequently, the aberrant spindle formation observed may result in the misalignment of sister chromosomes and subsequent cell mitosis failure. It is widely acknowledged that STAT3 plays a crucial role in various cellular processes such as cell proliferation, differentiation, and apoptosis. Previous studies have demonstrated that excessive activation of STAT3 contributes to the advancement of BLCA 31, 32. Consequently, we conducted a further investigation into the protein expression profile of the STAT3 signaling pathway in both the si-NC group and the si-CENPW group. However, our findings indicated that the knockdown of CENPW resulted in a decrease in the phosphorylation protein expression of STAT3 in 5637 and UM-UC-3 cells. The potential relationship between the interference of CENPW expression and the inhibition of bladder cancer may be attributed to the inactivation of STAT3. Ultimately, the tumor formation capacity of sh-CENPW cells was examined in vivo. It was observed that the subcutaneous tumor growth was comparatively slower in the sh-CENPW group in comparison to the sh-NC group. Furthermore, the Tunel assay revealed a higher occurrence of apoptosis in the tumors of the sh-CENPW group.

## Conclusion

In conclusion, our findings demonstrate a significant upregulation of CENPW in both BLCA tissues and cell lines. Moreover, a high expression of CENPW is associated with tumor progression in BLCA and predicts a poorer DDS. Notably, the inhibition of BLCA progression can be achieved through the knockdown of CENPW. Consequently, CENPW exhibits potential as an innovative therapeutic target and biomarker for prognostic prediction in BLCA. However, it is important to acknowledge certain limitations in this study. Firstly, the cohort sample size is relatively small and should be expanded in future research. Secondly, further investigation is required to elucidate the underlying mechanisms by which knockdown of CENPW arrests cell cycle and promotes apoptosis in BLCA cells.

## Supplementary Material

Supplementary figure and tables.
