# Supplementary material for: CENPW knockdown inhibits progression of bladder cancer through inducing cell cycle arrest and apoptosis
Source: J Cancer. 2024 Jan 1;15(3):858–70. doi: 10.7150/jca.90449 (PMC10777039; doi:10.7150/jca.90449)
Supplement: Supplementary file 1 — Supplementary figure and tables. [file jcav15p0858s1.pdf]

Supplementary Figure 1

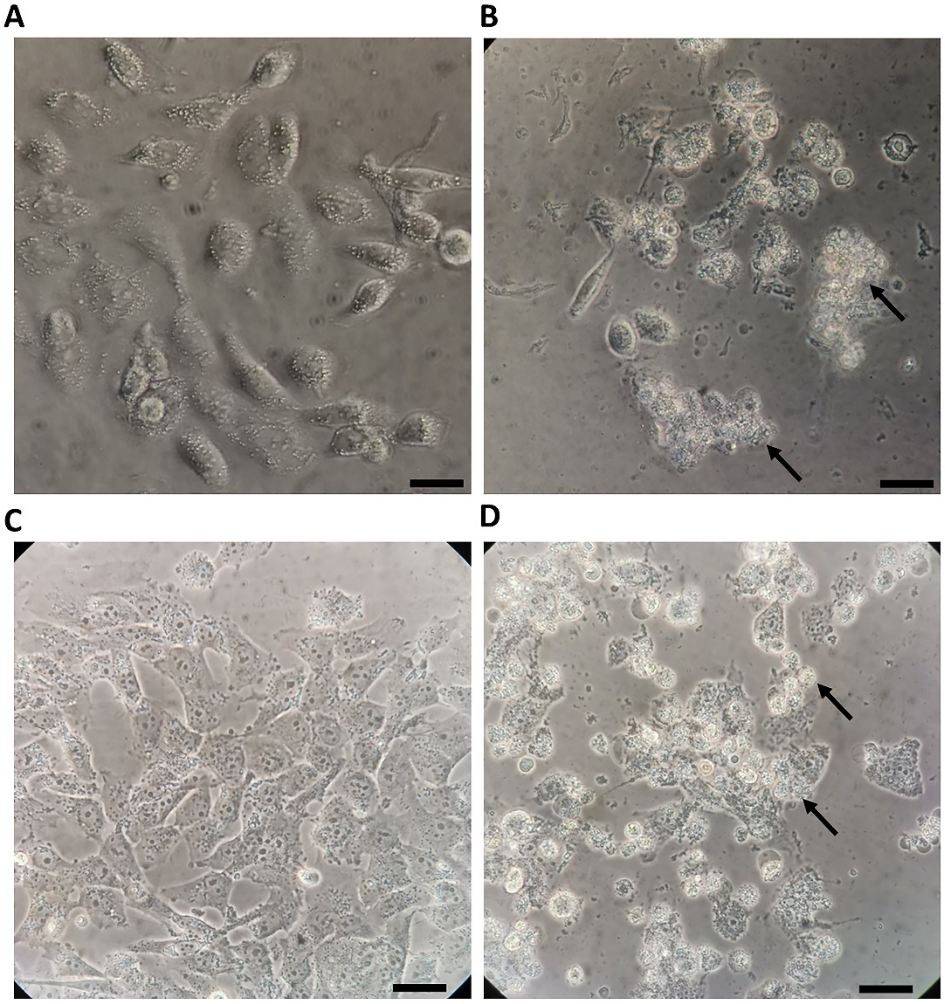

Figure S1. Cell morphological abnormalities (black arrow) in si-CENPW group of 5637 and UM-UC-3 cells for transfection siRNA 96hours. (A, C). si-NC group of 5637 and UM-UC-3 cells (B, D). si-CENPW group of 5637 and UM-UC-3 cells. Bars，50μm.

Supplementary Table 1. Clinicopathologic parameters in bladder cancer tissues (n=14).

ID	Gender	Age (year)	Tumor grade (WHO 2004)	Pathological stage
1	Male	72	low	Non-muscle-invasive
2	Male	81	low	Non-muscle-invasive
3	Female	69	High	Non-muscle-invasive
4	Female	48	High	Non-muscle-invasive
5	Female	73	High	Muscle-invasive
6	Female	80	High	Muscle-invasive
7	Male	66	High	Non-muscle-invasive
8	Male	78	High	Non-muscle-invasive
9	Male	69	low	Non-muscle-invasive
10	Male	77	High	Muscle-invasive
11	Female	27	low	Non-muscle-invasive
12	Male	80	High	Non-muscle-invasive
13	Male	70	low	Non-muscle-invasive
14	Male	68	High	Muscle-invasive

Supplementary Table 2. Primers used in this study

Name	Sequences (5'-3')
si-NC	UUCUCCGAACGUGUCACGUTT
si-CENPW	GCUUGUGCGAGUAAAUGUATT
shCtrl	GATCCGTTCTCCGAACGTGTCACGTAATTCAAGAGATTACGTGACACGTTCGGAGAATTTTTTC
shCENPW	GATCCGCTTGTGCGAGTAAATGTACTCGAGTACATTTACTCGCACAAGCTTTTTTG
β-actin-PCR-Forward	CCTGGCACCCAGCACAAT
β-actin-PCR-Reverse	GGGCCGGACTCGTCATAC
CENPW-PCR-Forward	ACCGGATTGTTTTCGCT
CENPW-PCR-Reverse	TCCGCTTTATCTGCTTCC
CDK1-PCR-Forward	AAAGTGAAGAGGAAGGGGTT
CDK1-PCR-Reverse	TGTACTGACCAGGAGGGATAG
CCNB1-PCR-Forward	TGGTTGATACTGCCTCTCC
CCNB1-PCR-Reverse	GACTGCTTGCTCTTCCTCA
PCNA-PCR-Forward	TAAGGGCCGAAGATAACG
PCNA-PCR-Reverse	TTCTCCTGGTTTGGTGCT
